# Enhanced Immunogenicity and Protective Effects against SARS-CoV-2 Following Immunization with a Recombinant RBD-IgG Chimeric Protein

**DOI:** 10.3390/vaccines12040356

**Published:** 2024-03-27

**Authors:** Mariângela de Oliveira Silva, Maria Fernanda Castro-Amarante, Alexia Adrianne Venceslau-Carvalho, Bianca da Silva Almeida, Isabela Pazotti Daher, Guilherme Antonio de Souza-Silva, Marcio Massao Yamamoto, Gabriela Koike, Edmarcia Elisa de Souza, Carsten Wrenger, Luís Carlos de Souza Ferreira, Silvia Beatriz Boscardin

**Affiliations:** 1 Laboratory of Antigen Targeting to Dendritic Cells, Department of Parasitology, Institute of Biomedical Sciences, University of São Paulo, São Paulo 05508-000, Brazil; mariangelasilva@usp.br (M.d.O.S.);; 2 Laboratory of Vaccine Development, Department of Microbiology, Institute of Biomedical Sciences, University of São Paulo, São Paulo 05508-000, Brazil; 3 Laboratory of Immunology, Heart Institute (InCor), School of Medicine, University of São Paulo, São Paulo 05403-000, Brazil; 4 Unit for Drug Discovery, Department of Parasitology, Institute of Biomedical Sciences, University of São Paulo, São Paulo 05508-000, Brazil

**Keywords:** SARS-CoV-2, subunit vaccines, recombinant RBD, IgG fusion

## Abstract

The unprecedented global impact caused by SARS-CoV-2 imposed huge health and economic challenges, highlighting the urgent need for safe and effective vaccines. The receptor-binding domain (RBD) of SARS-CoV-2 is the major target for neutralizing antibodies and for vaccine formulations. Nonetheless, the low immunogenicity of the RBD requires the use of alternative strategies to enhance its immunological properties. Here, we evaluated the use of a subunit vaccine antigen generated after the genetic fusing of the RBD with a mouse IgG antibody. Subcutaneous administration of RBD-IgG led to the extended presence of the protein in the blood of immunized animals and enhanced RBD-specific IgG titers. Furthermore, RBD-IgG immunized mice elicited increased virus neutralizing antibody titers, measured both with pseudoviruses and with live original (Wuhan) SARS-CoV-2. Immunized K18-hACE2 mice were fully resistant to the lethal challenge of the Wuhan SARS-CoV-2, demonstrated by the control of body-weight loss and virus loads in their lungs and brains. Thus, we conclude that the genetic fusion of the RBD with an IgG molecule enhanced the immunogenicity of the antigen and the generation of virus-neutralizing antibodies, supporting the use of IgG chimeric antigens as an approach to improve the performance of SARS-CoV-2 subunit vaccines.

## 1. Introduction

Responsible for the COVID-19 pandemic, the novel coronavirus 2 (SARS-CoV-2) emerged in Wuhan at the end of 2019 and rapidly spread across the world, impacting the economy and public health [1]. As of November 2023, the WHO has reported over 771 million confirmed cases and approximately 7 million deaths caused by COVID-19 [2]. The emergence of the pandemic highlighted the necessity to prioritize versatile and safe vaccine strategies. Fortunately, many vaccines based on diverse platforms have been developed and some were commercially available to prevent COVID-19, including mRNA vaccines, inactivated vaccines, viral vector vaccines and protein subunit vaccines [3,4,5,6]. Despite the significant global control of the COVID-19 pandemic, the continuous evolution of the virus and the emerging of variants remain a concern and require permanent vigilance and the development of vaccines that are safe and effective to induce protective immunity, capable of controlling the disease and neutralizing circulating viruses [7].

SARS-CoV-2 is a betacoronavirus, from the Coronaviridae family, and its genome encodes four structural proteins, including spike (S), nucleocapsid (N), envelope (E) and membrane (M) proteins [8,9]. In the course of infection, SARS-CoV-2’s entry into the host cell is mediated by the S protein, which binds to the human angiotensin-converting enzyme 2 (ACE2) using its receptor-binding domain (RBD) [9,10,11]. While the RBD has been shown to be the major target for neutralizing antibodies, it is worth noting that it presents suboptimal immunogenicity [12,13]

Various studies have proposed different strategies to enhance the effectiveness of RBD-based vaccines. These include utilizing the RBD in dimeric [14,15] and trimeric forms [16,17,18], as well as incorporating it into nanoparticle systems [19,20]. These approaches aim to improve the immunogenic response to the RBD, particularly by inducing virus-neutralizing antibodies circulating in the blood, thereby ultimately improving the efficacy and safety of anti-SARS-CoV-2 vaccines. In this context, the IgG fusion approach also presents crucial features to increase antigen immunogenicity, offering enhancements in its stability, persistence in the bloodstream, and uptake by antigen-presenting cells [21,22]. Although most studies have focused on Fc-fusion fragments, the full IgG fusion strategy has received less attention. While its application has been explored against viral infections [23], the available findings on this approach remain limited, particularly concerning SARS-CoV-2 infection.

To overcome the limited immunogenicity of subunit vaccines containing recombinant purified SARS-CoV-2 RBD, we genetically fused the sequence of the original virus (Wuhan Hu-1 strain) to the heavy chain of a mouse IgG1 antibody and produced the RBD-IgG protein by transient transfection. Our approach enhanced the stability of the RBD in the bloodstream and led to an enhanced protective immunity with regard to the non-fused RBD. Results based on neutralization of pseudotyped viral particles and in vivo challenges with SARS-CoV-2 virus particles demonstrated that the method represents a promising alternative for the generation of safe and effective anti-SARS-CoV-2 subunit vaccines based on recombinant proteins.

## 2. Materials and Methods

### 2.1. Construction and Preparation of Recombinant Proteins

The RBD-IgG construct was created by genetically fusing the Wuhan Hu-1 RBD gene (derived from plasmid pCAGGS-RBD, kindly provided by Dr. Florian Krammer, Icahn School of Medicine at Mount Sinai) [24] to a mouse IgG1 at the C-terminal end of the heavy chain [25]. This IgG1 heavy chain carries a D265A mutation that abolishes binding to mouse Fc receptors [26]. The plasmids encoding the heavy and the light chains of the monoclonal antibody (originally derived from the GL117 clone with specificity for the bacterial beta galactosidase) were kindly provided by Dr. Michel C. Nussenzweig (The Rockefeller University). The fusion antibody was expressed utilizing the Expi293^TM^ system (Thermo Fisher Scientific, Waltham, MA, USA), and subsequently purified through protein G (GE Healthcare, Chicago, IL, USA) affinity chromatography, exactly as described elsewhere [27]. The Beta, Gamma, Delta, and Omicron (BA.2 variants) RBD constructs were synthesized commercially, employing the point mutations previously described [28]. The recombinant RBD proteins were produced according to the protocol described by Stadlbauer et al. [29]. Briefly, Expi293F^TM^ cells were transfected using the pCAGGS-RBD plasmid and purified by Ni-NTA resin (Invitrogen, Waltham, MA, USA). The purity of the recombinant proteins was determined by 12% SDS–PAGE and visualized by staining with Coomassie blue dye (VWR Chemicals, LLC, Radnor, PA, USA).

### 2.2. Immunization and Protection Experiments

Five-six week-old K18-hACE2 transgenic mice (JAX stock #034860) [30] were obtained from an isogenic mouse facility of the Institute of Biomedical Sciences II, University of São Paulo, Brazil. The animal handling procedures employed in this study are in compliance with the guidelines set forth by the Institutional Animal Care and Use Committee (IACUC) of the Institute of Biomedical Sciences at the University of São Paulo. These procedures received official approval from the IACUC under the reference number 5625240522. All experiments conducted adhered to the principles outlined in the NIH Guide for the Care and Use of Laboratory Animals and were carried out in accordance with the regulations stipulated in the Brazilian National Law on animal care (11.794/2008).

The K18-hACE2 transgenic mice were immunized with two doses of 5 μg of RBD-IgG plus 50 μg of Poly (I:C) (HMW, InvivoGen, San Diego, CA, USA), as adjuvant by the subcutaneous route (SC), 14 days apart. As control groups, the animals received an equimolar amount of non-fused RBD (1.4 μg) plus Poly (I:C) or Poly (I:C) alone. Two independent experiments (n = 5–6 animals/group) were performed. For immunogenicity analysis, blood samples were collected on days 14 and 28 after the last dose, and serum samples were obtained by centrifugation.

To conduct the lethal challenge experiments, 15 days after the administration of the second dose, the animals were intranasally infected with 2.8 × 10^5^ TCID_50_ of the SARS-CoV-2 Wuhan strain (GenBank MT350282.1) in 30 μL of DMEM. The challenge was carried out under anesthesia using isoflurane. Mice were monitored daily for disease signs. The human endpoint was determined by the presence of clinically diagnostic signs of respiratory distress, which included severe respiratory difficulties, as well as a weight loss exceeding 25%. In cases where mice exhibited severe symptoms or exceeded the defined weight-loss threshold, they were euthanized using an anesthesia overdose. At 6 days post-infection (dpi), all surviving mice were euthanized, and their lungs and brains were collected for further analysis. Viral infections were conducted within a Biosafety Level 3 (BSL-3) facility at the Institute of Biomedical Sciences.

### 2.3. Plasma Persistence of the Recombinant Protein

A total of 35.7 μg of purified RBD-IgG and 10 μg of RBD proteins were administered subcutaneously to C57BL/6 mice (n = 3 per group). Serum samples were collected at 2, 24, 48, 72, 96, 120, and 144 h post-administration. The protein concentrations in the pooled blood circulation were quantified by ELISA with a human monoclonal antibody against the RBD (developed in-house). Known concentrations of RBD-IgG and RBD proteins were used to establish a standard curve at OD_490_.

### 2.4. Pseudovirus Production and Neutralization Assays

Pseudoviruses expressing a full-length spike protein from the Wuhan Hu-1 strain were produced as previously described [31]. Briefly, HEK293T cells were co-transfected using plasmids encoding firefly luciferase (pNL43R-E-luciferase) and the SARS-CoV-2 Wuhan S protein (pcDNA.3), kindly provided by Dr. Paul D. Bieniasz (The Rockefeller University). After 48 h, pseudovirus-containing supernatants were collected, centrifuged, and stored at −80 °C. Viral titers were measured by their luciferase activity in relative light units. For the neutralization assay, HT1080-hACE2 cells (kindly provided by Dr. Paul D. Bieniasz, The Rockefeller University) were seeded with a density of 1 × 10^4^ cells/well in 96-well plates (Corning, New York, NY, USA) and incubated at 37 °C for 24 h. Serial dilutions of heat-inactivated serum samples (56 °C for 30 min) were incubated with pseudoviruses for 1 h at 37 °C. After the incubation, the mixtures were added to the pre-seeded cells and incubated at 37 °C for 48 h. The luminescence emission was measured using Luciferase Assay System kits (Promega, Madison, WI, USA). The IC_50_ was defined as the reciprocal of the serum dilution at which the relative light units (RLU) decreased by 50% compared to the virus control wells. The neutralization assay was performed using pooled sera from two independent immunization experiments (n = 5 animal/group)

### 2.5. Live Virus-Neutralizing Antibody Assay

The efficacy in neutralizing the live SARS-CoV-2 virus was evaluated via a cytopathic effect-based virus neutralization test (CPE-VNT), employing the established protocol as detailed by Mendrone-Junior et al., 2021 [32]. In summary, heat-inactivated serum samples were serially diluted in a DMEM containing 2% Fetal Bovine Serum (FBS). The dilutions’ range extended from 1:20 to 1:2560, with a 2-fold dilution factor. Each diluted serum sample was duplicated and mixed with the SARS-CoV-2/human/BRA/SP02cc/2020 virus strain (GenBank accession number: MT350282.1) at a concentration of 10^3^ TCID_50_/mL to achieve a final virus concentration of 100 TCID_50_. Next, mixtures were incubated at 37 °C for 1 h. After the incubation period, 100 μL of the mixture was added to Vero cells (ATCC CCL-81), previously seeded at a density of 1 × 10^5^ cells per well in 96-well plates. The plates were further incubated at 37 °C with 5% CO_2_ for 72 h. The observation of the cytopathic effect was carried out visually under microscopic examination. The highest serum dilution level that completely prevented virus proliferation, as indicated by the absence of cytopathic effects, was identified as the 100% neutralization titer (VNT_100_). Importantly, all experiments involving SARS-CoV-2 were conducted within a Biosafety Level 3 (BSL-3) facility at the Institute of Biomedical Sciences.

### 2.6. Enzyme-Linked Immunosorbent Assays (ELISA)

To evaluate if the RBD antigenicity was retained after the fusion with the IgG1 molecule, 96-well plates (Costar) were coated with 100 ng/well of the purified RBD-IgG fusion or an equivalent amount of the RBD non-fused and incubated overnight at room temperature (RT). The following day, the plates were washed three times with a phosphate-buffered saline (PBS) solution containing 0.02% Tween-20 (PBS-T) and blocked with a solution containing 5% non-fat milk and 1% bovine serum albumin (BSA) in PBS-T. Serially diluted human sera from vaccinated and convalescent individuals were added and incubated at 37 °C for 2 h. Secondary HRP-conjugated goat anti-human IgG antibody (Invitrogen, catalog number: A18823) was added, and the plate was incubated for 1 h at RT. The absorbance of each well at 492 nm was measured using an ELISA plate reader (BioTek, Winooski, VT, USA).

To measure the anti-RBD antibodies induced after immunization, serum samples were collected from mice 14 days after each dose via submandibular bleeds. We assessed the levels of RBD-specific IgG, IgG1, IgG2b, IgG2c and IgG3 via ELISA following the procedure described above. In brief, the recombinant RBD (100 ng/well) was used to coat 96-well plates. After blocking, serially diluted mouse sera were incubated for 2 h at 37 °C. This step was followed by the incubation of the plates with goat anti-mouse IgG, IgG1, IgG2b, IgG2c or IgG3 antibodies conjugated with horseradish peroxidase (HRP) for 1 h at RT. Anti-RBD antibody titers were normalized in a log10 scale considering the highest serum dilution showing an OD_492_ > 0.1.

### 2.7. Quantification of Viral Load by RT-qPCR

The viral loads in the lungs and brain were measured by determining the RNA copies/gram of tissue, and then calculated as TCID_50_/mL/g of tissue. In brief, the tissues were collected, individually weighted, and then added to tubes containing 500 μL of DMEM supplemented with 1% penicillin-streptomycin, and 1 mm of glass beads. Next, the tubes were homogenized using Qiagen’s TissueLyser II at 30 Hz twice for 2 min, followed by centrifugation at 2000× *g* for 30 sec. Supernatants were used for total RNA extraction using the MagMAX™ Viral/Pathogen II (MVP II) Nucleic Acid Isolation Kit (Thermo Fisher scientific). SARS-CoV-2 RNA detection was performed according to a protocol described previously to amplify the SARS-CoV-2 E gene (probe: FAM-ACACTAGCCATCCTTACTGCGCTTCG-BQ, primers: F 5′ ACAGGTACGTTAATAGTTAATAGCGT 3′ and R 5′ ATATTGCAGCAGTACGCACACA 3′) [33]. The brain and lung samples were processed in duplicate using the AgPath-ID™ One-Step RT-PCR Reagent (Thermo Fisher, Waltham, MA, EUA) and the results were expressed as TCID_50_/mL/g of tissue.

### 2.8. Statistical Analysis

We used Prism 9.0 (GraphPad, San Diego, CA, USA) for all the analyses. A one-way ANOVA was used for multiple comparisons, followed by Tukey’s multiple comparison post-test for the comparison of specific groups. *p* < 0.05 was considered significant. A power analysis was not performed.

## 3. Results

### 3.1. Generation of the Recombinant RBD-IgG Antigen, Antigenicity and Persistence in the Blood

**Figure 1:**
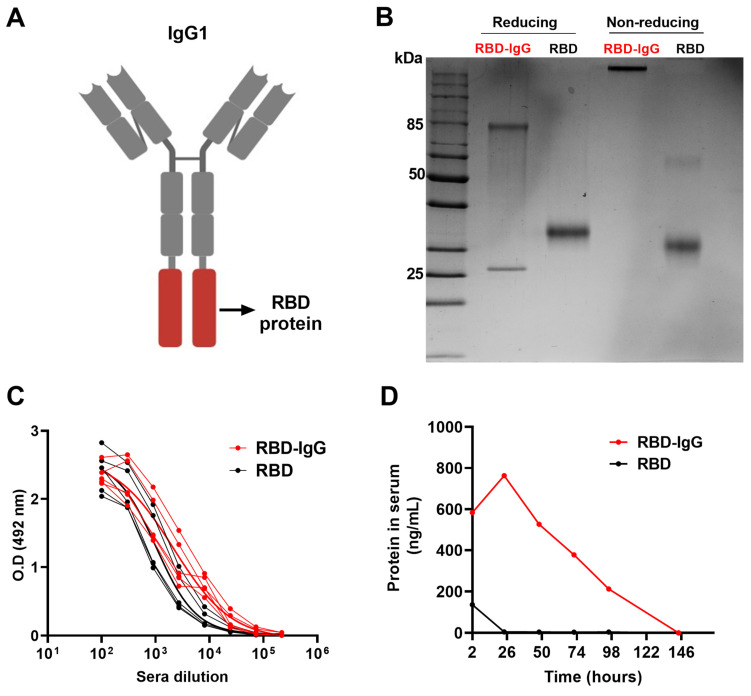
Purification and characterization of the chimeric RBD-IgG antigen. (**A**) Structural representation of the chimeric RBD-IgG molecule. (**B**) SDS-PAGE of RBD-IgG and non-fused RBD proteins under both reducing and non-reducing conditions. (**C**) Assessment of RBD-IgG antigenicity via testing with sera from five COVID-19 convalescents and vaccinated individuals. (**D**) Persistence of the recombinant proteins in the blood of mice following subcutaneous administration. Equimolar amounts of the purified RBD-IgG (35.7 μg) and non-fused RBD proteins (10 μg) were subcutaneously inoculated into K18-hACE2 mice. The protein concentrations in pooled sera were measured via ELISA.

After the expression of the recombinant RBD-IgG antigen (Figure 1A) in Expi293F^TM^ cells and purification through affinity chromatography, we conducted a characterization of the protein under both reducing and non-reducing conditions using SDS-PAGE and Western blot (Figure 1B). As shown in Figure 1B, two bands with molecular weights of 80 kDa and 25 kDa, under reducing conditions, and a single band at approximately 210 kDa, under non-reducing conditions, were detected in polyacrylamide gels. Western blot analysis employing the anti-RBD and anti-IgG monoclonal antibody confirmed the identity of the RBD antigen and heavy and light chains bands (Supplementary Figure S1). These results suggest that RBD-IgG was successfully expressed and assembled in the human cells. To assess the antigenicity of the chimeric antigen, we tested the reactivity with human serum samples collected from individuals previously infected with the SARS-CoV-2 and vaccinated with an anti-COVID-19 vaccine. The results in Figure 1C suggest that the genetic fusion of the molecules did not compromise the antigenicity of the RBD. In fact, the RBD fused to IgG was more readily recognized (*p* < 0.0317) by the sera from individuals previously infected or vaccinated compared to the non-fused RBD. This discrepancy in recognition could potentially be attributed to alterations in the exposure of epitopes on the protein following fusion.

Previous evidence indicated that the fusion of antigens with IgG fragments could be an important immunopotentiator to enhance the stability of the chimeric antigen after administration [34]. Thus, we evaluated the persistence of the purified RBD and RBD-IgG after subcutaneous administration to mice. For that purpose, blood samples of the inoculated mice were collected daily for a period of one week and the amount of antigens in the blood were measured via RBD-specific ELISA. As demonstrated in Figure 1D, the chimeric antigen was detected in blood samples up to 98 h (four days) after inoculation, whereas the non-fused RBD persisted less than 24 h after parenteral administration to the mice. This result clearly demonstrated that the fusion of the target antigen (RBD) to an IgG molecule enhanced the stability and persistence of the chimeric protein in the blood of inoculated animals.

### 3.2. RBD Fused to IgG Induces Strong Anti-Viral Antibody Responses

**Figure 2:**
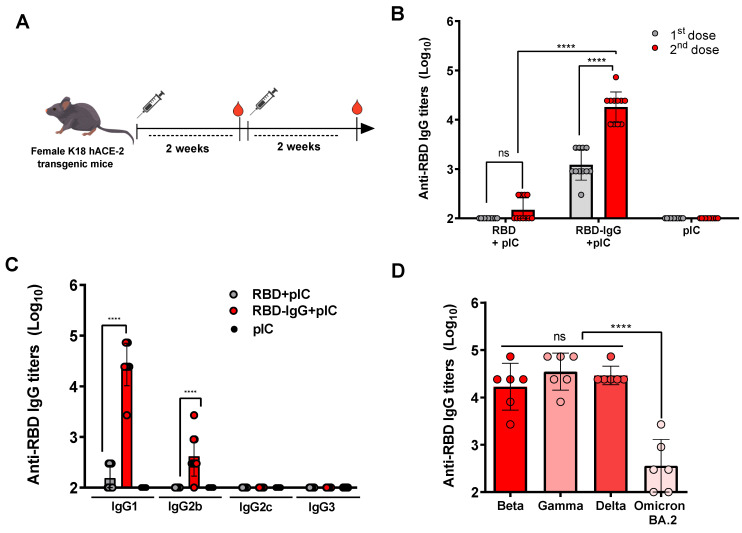
RBD-specific IgG responses elicited among immunized mice. (**A**) Schematic representation of the immunization regimen. Five-to-six week-old K18-hACE2 mice were immunized twice with 5 μg of RBD-IgG or 1.4 μg of the non-fused RBD, both adjuvanted with 50 μg of Poly (I:C), administered at a 2-week interval. Sera were collected 14 days after each dose, and detected for specific IgG and subclass antibodies. (**B**) Serum SARS-CoV-2 RBD-specific IgG titers were measured via ELISA 14 days after each immunization. (**C**) Serum anti-RBD IgG1, IgG2b, IgG2c and IgG3 subclass titers after the second dose. (**D**) Cross-reactivity of antibodies elicited in mice immunized with RBD-IgG against different SARS-CoV-2 variants of concern. Bars show mean ± SD from two representative experiments (n = 5–6 animals/group) in (**B**,**C**) and from one representative experiment (n = 6 animals/group) in (**D**). Statistical differences are indicated in the graphs, **** *p* < 0.0001. One-way ANOVA with Tukey’s post-test.

To determine the impact of the genetic fusion approach on the immunogenicity of the target antigen, mice received two SC injections with equimolar amounts of the RBD-IgG or non-fused RBD, in the presence of Poly (I:C) as an adjuvant (Figure 2A). As indicated in Figure 2B, the results revealed that mice immunized with RBD-IgG elicited a significantly higher anti-RBD serum IgG response in comparison to mice immunized with the non-fused RBD. Notably, mice receiving RBD-IgG exhibited detectable titers (>10^3^) of anti-RBD IgG even after the first immunization and reached maximal titers (average 5 × 10^4^) two weeks after the second dose. In contrast, in the same experimental conditions, mice immunized with the non-fused adjuvanted with Poly (I:C) showed maximum antigen-specific serum IgG titers of approximately 10^2^ two weeks after the second immunization. To gain further insight into the nature of the immune responses induced after vaccination with the purified antigens, we conducted IgG subclass analyses of the serum samples collected from mice submitted to the different immunization groups. Following the administration of two doses of RBD-IgG, a marked increase in IgG1 antibodies specific to the RBD protein was observed. Anti-RBD IgG2b titers were also detected at comparatively lower values (Figure 2C). In contrast, IgG1 was the only subclass detected, at a rather low level, in blood samples collected from mice immunized with the purified RBD. These findings indicate that the RBD-IgG immunization preferentially stimulates the production of IgG1 antibodies, suggesting a Th2-type biased immune response profile.

Additionally, we explored whether the RBD-IgG vaccine formulations would a induce cross-reactive antibody response against different variants of concern of the SARS-CoV-2 virus. For this purpose, serum samples from the tested immunization groups were collected 14 days after the last dose, and subsequently tested for their reactivity in an ELISA with the purified recombinant RBD proteins derived from the Beta, Gamma, Delta and Omicron (BA.2) SARS-CoV-2 variants. As illustrated in Figure 2D, the sera collected from mice immunized with the RBD-IgG effectively bound to the recombinant proteins derived from the Beta, Gamma, and Delta variants (average antibody titer: 4.22 log10, 4.54 log10 and 4.46 log10, respectively) without any significant differences observed among the tested antigens. There was also a detectable reactivity to the RBD protein derived from the Omicron BA.2 variant (average antibody titer: 2.55 log10), but the reaction was drastically reduced with regard to the values detected from the other SARS-CoV-2 variants.

### 3.3. Immunization with the Chimeric RBD-IgG Enhances the Induction of SARS-CoV-2-Neutralizing Antibodies

**Figure 3:**
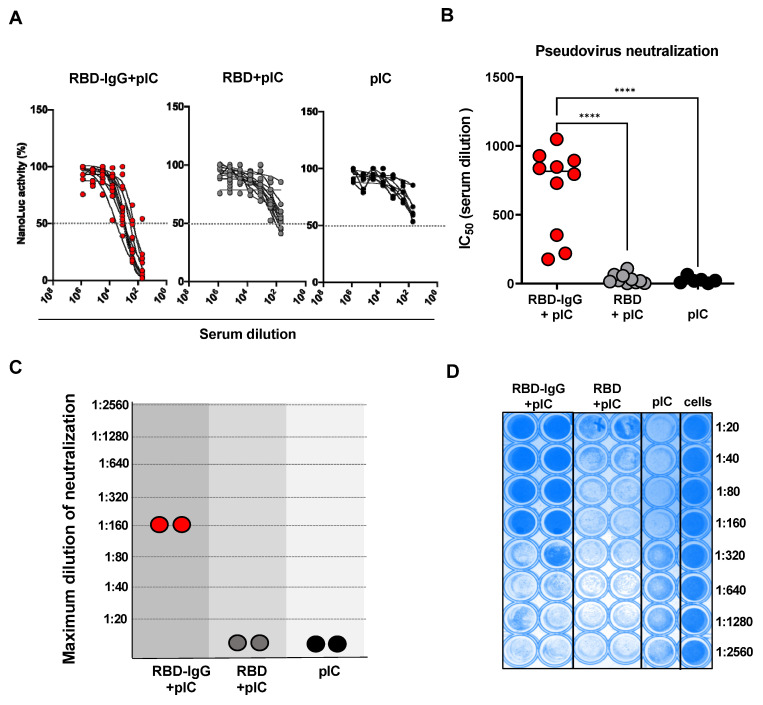
Neutralizing antibody responses elicited in mice immunized with RBD-IgG. Five-to-six week-old K18-hACE2 transgenic mice were immunized according to the immunization regimen depicted in Figure 2. (**A**) Individual values of the neutralizing activity of individually tested serum samples using the pseudovirus prepared with the S protein derived from the SARS-CoV-2 Wuhan strain. (**B**) The IC_50_ values were determined by NanoLuc activity using sigmoidal nonlinear regression analyses. Data were collected from two independent experiments (n = 10). (**C**) Maximum dilution of sera after the second dose of the RBD-IgG vaccine capable of neutralizing live Wuhan SARS-CoV-2 particles by VNT_100_. (**D**) Neutralization plate with serum samples from immunized mice. Wells with strong blue staining indicate complete neutralization or non-infected cells. Pooled sera from two independent experiments were used for the analysis (n = 10 animals/group). Statistical differences are indicated in the graphs, **** *p* < 0.0001. One-way ANOVA followed by Tukey’s post-test.

We proceeded to determine whether the anti-RBD antibodies elicited in mice after immunization with RBD-IgG or the non-fused RBD could effectively neutralize virus particles. For that purpose, we applied two different experimental approaches: in vitro neutralization tests carried out with recombinant pseudovirus particles and in vivo tests performed after challenging vaccinated mice with infective Wuhan SARS-CoV-2 particles. The in vitro tests employed serum samples collected after the second dose and individually tested against the pseudovirus made with the Wuhan S protein. As shown in Figure 3A,B, immunization with chimeric RBD-IgG induced high levels of SARS-CoV-2-neutralizing antibodies when compared to mice immunized with the non-fused RBD or inoculated only with the adjuvant.

To further validate the virus neutralization effects induced by the tested vaccine antigens, we also determined whether the serum samples could inhibit the infectivity of live SARS-CoV-2 particles. For that purpose, pooled serum samples of each tested group were diluted and mixed with the SARS-CoV-2 virus particles of the Wuhan strain. As shown in Figure 3C, only sera collected from mice immunized with RBD-IgG contained antibodies capable of efficiently neutralizing the infectivity of the SARS-CoV-2 virus (100% virus neutralization at a dilution of 1:160 of the serum pool). These results were evident by the absence of the cytopathic effect in infected Vero cells (dark blue), as can be seen in Figure 3D.

### 3.4. Immunization with the Chimeric RBD-IgG Confers Protective Immunity to Lethal Challenge with SARS-CoV-2

**Figure 4:**
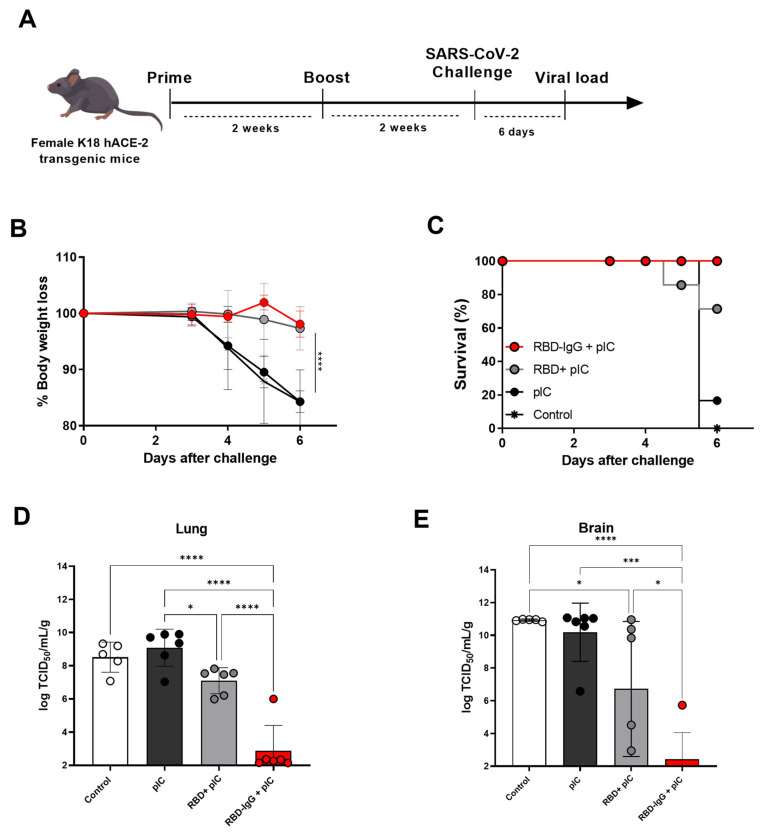
Protective efficacy of immunization with RBD-IgG to lethal challenges with SARS-CoV-2. (**A**) K18-hACE2 mice were immunized according to the immunization regimen described in Figure 2. On day 28 after the first dose, mice were challenged intranasally with 2.8 × 10^5^ TCID_50_ of the Wuhan SARS-CoV-2 strain. (**B**) Body weight loss. (**C**) Survival. (**D**) Viral load detection via RT-qPCR in the lungs and (**E**) brains of mice infected on the day of death (six days after the infection). Data are expressed as the mean ± SD from one representative experiment (n = 5–6 animals/group). Statistical differences are indicated in the graphs, **** *p* < 0.0001, *** *p* < 0.001, * *p* < 0.05. One-way ANOVA followed by Tukey’s post-test.

We next evaluated the protective effects conferred by immunization with RBD-IgG against a lethal challenge of SARS-CoV-2. For that purpose, susceptible K18-hACE2 mice were immunized with two doses of RBD-IgG admixed with Poly (I:C). Two weeks after the second dose, sera RBD-IgG titers were examined (Supplementary Figure S2) and mice were subjected to a lethal challenge with the SARS-CoV-2 Wuhan strain through intranasal inoculation with 2.8 × 10^5^ TCID_50_ (Figure 4A). Throughout a six-day observation period, mice were closely monitored for clinical signs including weight loss, eye closure, activity levels, and overall appearance. Euthanasia was performed when animals reached a clinical score considered to be the endpoint. Following the challenge, all RBD-IgG immunized mice did not present significant weight loss (Figure 4B) and showed 100% survival at the end of the observation period (Figure 4C). Mice immunized with the non-fused RBD did not show significant weight loss after the challenge with the SARS-CoV-2 and 75% of the vaccinated mice survived at the end of the observation period. Analysis of the viral loads in the lungs (Figure 4D) and brains (Figure 4E) of the challenged mice showed a drastic reduction of the virus load in mice immunized with RBD-IgG. In contrast, mice immunized with the RBD had a significantly higher value of replication copies of the virus genome, both in the lungs and brain, with regard to the group immunized with the chimeric protein.

## 4. Discussion

The last three years have witnessed an unprecedented advancement of vaccines based on different technological platforms. The impact of the COVID-19 pandemic promoted enormous investments in the development of vaccine candidates and huge revenues for companies capable of obtaining approval for their use in humans, particularly those based on mRNA and adenovirus-vectorized formulations [3,35]. Nonetheless, the global need for effective and safe vaccines capable of coping with emerging SARS-CoV-2 variants and other potential pandemic-level infectious agents demands continuous research efforts that could lead to new formulations and technologies [36]. In this study, we evaluated an experimental approach capable of improving the performance of subunit vaccines based on recombinant proteins. For this purpose, we investigated the immunological performance of a COVID-19 subunit vaccine based on the fusion of a recombinant form of the SARS-CoV-2 spike protein, encompassing the region responsible for the entrance of the virus into host cells (the RBD region), with a mouse IgG molecule. Our findings demonstrated that the resulting chimeric protein did not affect the antigenicity but significantly enhanced the immunogenicity of the original RBD protein, leading to higher antigen-specific serum antibody (IgG) responses, virus-neutralizing antibodies and protection in mice submitted to a lethal challenge with the original (Wuhan) SARS-CoV-2 strain. Altogether, the evidence gathered in this study indicated that the fusion of antigens to an IgG molecule has the potential to improve the immunological performance of the antigens and, therefore, contribute to the development of alternative vaccine formulations against SARS-CoV-2 based on a rather simple and straightforward technological approach.

The notable advantages of subunit vaccines are their safety and rather straight production pipelines [37,38]. However, such vaccines often fail to elicit strong and durable immune responses, often requiring the use of high doses and/or repeated immunizations [39]. In the context of COVID-19 vaccine development, much attention has been focused on the RBD protein as the primary immunogen. Since the RBD alone has limited immunogenicity and induces low levels of neutralizing antibodies [40], alternative strategies have been explored to increase its immunogenicity, such as its incorporation into nanoparticles, and its combination with immunomodulatory compounds, among others [19,41,42]. The fusion of antigens to the Fc portion of an IgG molecule has been repeatedly demonstrated to increase the immunogenicity of antigens both in mice and other mammal species, the recovery yields and stability of the chimeric proteins [43,44,45,46]. However, our current findings suggest that an alternative strategy, involving the fusion of antigens to the C-terminal end of the entire IgG molecule, can also improve immunogenicity and antigen stability for the target antigen. Differing from the conventional approach based on Fc fragment fusion, here we fused an entire mouse IgG molecule to the RBD protein from the SARS-CoV-2 Wuhan strain. Our IgG construct carries a specific mutation (D265A) in the constant region that interferes with FcR binding [26] and extends the presence of the antigen in the bloodstream [47], probably enhancing the interaction events with antigen-presenting cells. Indeed, our experiments demonstrated that mice immunized with the chimeric antigen showed extended antigen persistence in the serum compared to the non-fused RBD.

The effective induction of neutralizing antibodies by vaccines usually requires the maintenance of the native structural conformation of antigen epitopes. In this context, we showed that human sera from SARS-CoV-2-infected patients and Pfizer-immunized individuals recognized the recombinant RBD-IgG antigen. Such results demonstrate that the fusion with the IgG molecule did not alter the antigenicity of the RBD and, thus, indicate that, at least partially, the conformation of the RBD moiety in the fusion protein has not been altered. RBD-specific antibodies from human sera generated during natural infection and/or after vaccination exhibited reactivity with both the RBD-fused IgG and non-fused RBD antigens, demonstrating that the IgG fusion strategy preserved conformational features of the viral antigen.

Furthermore, we explored the immunological features of the RBD-IgG antigen using a two-dose immunization regimen. Our findings revealed that RBD-IgG induced significantly higher antibody titers compared to the non-fused-RBD and, thus, conferred an adjuvant effect to the original antigen leading to enhanced immunogenicity. Additionally, sera from RBD-IgG-immunized mice exhibited stronger cross-reactivity with SARS-CoV-2 antigens derived from other variants of concern, such as Beta, Gamma, and Delta. Given the crucial role of neutralizing antibodies in a vaccine’s protective effects [48], we also employed the SARS-CoV-2 pseudovirus assay to assess the neutralization activity of the individual anti-RBD serum generated after the second dose. Notably, antibodies raised in RBD-IgG-immunized mice neutralized spike-expressing pseudovirus, whereas sera from non-fused RBD-immunized mice showed reduced neutralization activity. To further validate these findings, we conducted a cytopathic effect-based virus neutralization test using the SARS-CoV-2 Wuhan strain to determine the virus-neutralizing effect of the induced antibody responses. Sera from RBD-IgG-immunized mice showed significant neutralization titers (up to 160), while sera collected from RBD-immunized mice did not exhibit a significant virus neutralization effect (titers < 20). Collectively, our results indicate the potential of IgG fusion as an approach to enhance the immunogenicity of the RBD and to generate neutralizing antibodies.

K18-hACE2 has been frequently used as a model to test the effectiveness of vaccines in providing immunity against SARS-CoV-2. This genetically modified mouse strain develops lung infections that closely resemble severe cases of human COVID-19 [49]. In our study, K18-hACE2 mice were challenged with the Wuhan strain, and their morbidity, weight loss and virus titer in their lungs were measured as parameters to evaluate vaccine efficacy. The post-challenge analysis revealed that the RBD-IgG-immunized group did not experience significant body weight loss over time, and all animals were protected from the lethal challenges with SARS-CoV-2. Furthermore, the virus titers in the lungs and brains were almost undetectable. Notably, despite protection observed in mice immunized with the non-fused RBD, such mice showed elevated viral loads after challenge in both their lungs and brain.

One distinctive aspect of an IgG fusion vaccine platform is its versatility and rather easy and fast adaptation to different antigens, such as the RBD expressed by Omicron variants, which are known to escape the immunity induced by previously circulating SARS-CoV-2 variants. Moreover, fusion with IgG facilitates simple large-scale purification via affinity chromatography, resulting in immunogens with an enhanced stability at different temperatures and, thus, an enhanced shelf life [50], unlike mRNA vaccines that require storage at much lower temperatures.

Another interesting aspect of fusing an antigen to a full IgG is the ability to engineer mAbs that bind to specific cell surface molecules, thereby directing the antigen or drug to a particular cell of interest. This strategy has been explored using mAbs targeted at various immune cell surface receptors or tumor cells [51,52]. Here, we utilized an mAb with specificity for the bacterial beta galactosidase (i.e., not binding to any mouse cell or tissue), hence, not addressing whether targeting the RBD to an antigen-presenting cell would enhance the construct’s immunogenicity.

Finally, it is worth mentioning that the strategy described here generated a full IgG antibody fused to the antigen of interest. This is different from the more commonly described strategies that use antigens fused to an IgG Fc portion [43,44,45,46,53]. Based on the results described here, this strategy is also able to improve the immunogenicity of the fused protein. Nonetheless, more experiments designed to compare, under identical experimental conditions, the immunogenicity of RBD-IgG and RBD-Fc fusion are still necessary.

## 5. Conclusions

In summary, the present study supports the use of a full-length monoclonal antibody fused to an antigen of interest to improve immunogenicity and adds an alternative to the technological repertoire presently available for the development of safe and effective anti-virus vaccines.

## Data Availability

Data are contained within the article and Supplementary Materials.

## Supplementary Materials

The following supporting information can be downloaded at: https://www.mdpi.com/article/10.3390/vaccines12040356/s1, Figure S1: Detection of RBD and IgG chains in Western blot analysis; Figure S2: Serological analysis of RBD-IgG titers in pre-challenged mice.
