# Supplementary material for: Enhanced Immunogenicity and Protective Effects against SARS-CoV-2 Following Immunization with a Recombinant RBD-IgG Chimeric Protein
Source: Vaccines (Basel). 2024 Mar 27;12(4):356. doi: 10.3390/vaccines12040356 (PMC11054318; doi:10.3390/vaccines12040356)
Supplement: Supplementary file 1 [file vaccines-12-00356-s001.zip › vaccines-2863631-supplementary.pdf]

Supplementary material

1. Methods

1.1-*Western blot analysis*

Protein samples, comprising 2 µg of purified RBD-IgG and 0.56 µg of non-fused RBD proteins, underwent polyacrylamide gel electrophoresis (SDS-PAGE, 12%) under both reducing and non-reducing conditions. Subsequently, the separated proteins were transferred onto nitrocellulose membranes (GE Healthcare Life Sciences). Membrane staining was performed with a human monoclonal anti-RBD (1µg/mL) (produced in house) or with a mouse anti-Kappa and anti-IgG Fc-specific antibodies. Reactive protein bands were identified by exposure of the membranes to a luminol-hydrogen peroxide solution according to the manufacturer's instructions (Sigma Aldrich, San Luis, USA).

2. Figure

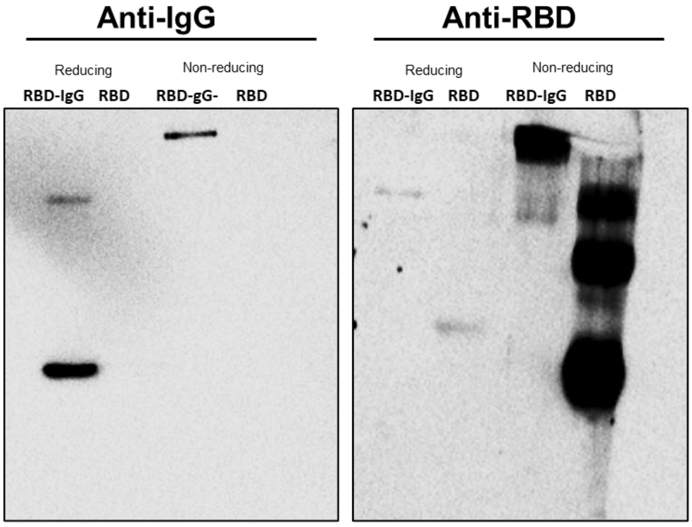

Figure S1. Detection of RBD and IgG chains in Western blot analysis. Aliquots containing 2 µg of purified RBD-IgG and 0.56 µg of non-fused RBD proteins were sorted in 12% polyacrylamide gels under both reducing and non-reducing conditions. Detection of IgG chains was performed with a mouse anti-Kappa and anti-IgG Fc-specific antibodies, and RBD with a human monoclonal anti-RBD antibody (1µg/mL).

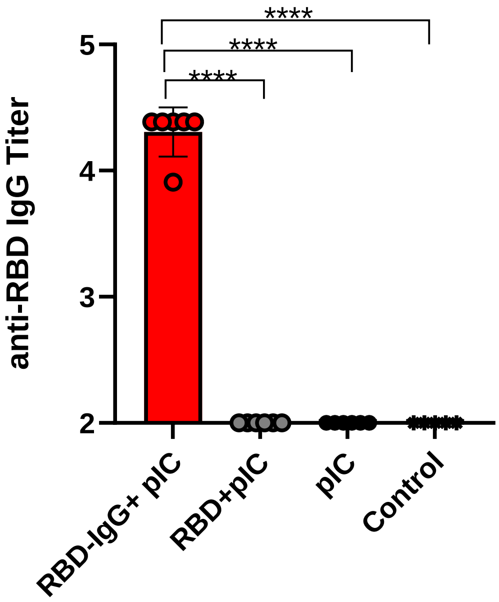

Figure S2. Serological analysis of RBD IgG titers in pre-challenged mice. K18-hACE2 mice were immunized twice with 5 μg of RBD-IgG or 1.4 μg of non-fused RBD, both adjuvanted with 50 μg of Poly (I:C), administered at a 2-week interval. Sera were collected 14 days after the last dose and detected for specific IgG. After that, the animals were challenged intranasally with 2.8×10^5 $TCID_{50}$ of the Wuhan SARS-CoV-2 strain, as described in Figure 4. Results represented as mean ± SD. Statistical differences are indicated in the graphs, ****$p < 0.0001$. One way ANOVA with Tukey's post-test
